# Cortical hierarchy disorganization in major depressive disorder and its association with suicidality

**DOI:** 10.3389/fpsyt.2023.1140915

**Published:** 2023-04-24

**Authors:** Lin Shiwei, Zhang Xiaojing, Zhang Yingli, Chen Shengli, Lin Xiaoshan, Xu Ziyun, Hou Gangqiang, Qiu Yingwei

**Affiliations:** ^1^Department of Radiology, Huazhong University of Science and Technology Union Shenzhen Hospital, Shenzhen, China; ^2^Guangdong Provincial Key Laboratory of Genome Stability and Disease Prevention and Regional Immunity and Diseases, Department of Pathology, Shenzhen University School of Medicine, Shenzhen, Guangdong, China; ^3^Department of Depressive Disorder, Shenzhen Kangning Hospital, Shenzhen Mental Health Center, Shenzhen, Guangdong, China; ^4^Department of Radiology, Shenzhen Kangning Hospital, Shenzhen Mental Health Center, Shenzhen, China

**Keywords:** major depressive disorder, suicide, resting-state fMRI, connectome gradient, stepwise connectivity

## Abstract

**Objectives:**

To explore the suicide risk-specific disruption of cortical hierarchy in major depressive disorder (MDD) patients with diverse suicide risks.

**Methods:**

Ninety-two MDD patients with diverse suicide risks and 38 matched controls underwent resting-state functional MRI. Connectome gradient analysis and stepwise functional connectivity (SFC) analysis were used to characterize the suicide risk-specific alterations of cortical hierarchy in MDD patients.

**Results:**

Relative to controls, patients with suicide attempts (SA) had a prominent compression from the sensorimotor system; patients with suicide ideations (SI) had a prominent compression from the higher-level systems; non-suicide patients had a compression from both the sensorimotor system and higher-level systems, although it was less prominent relative to SA and SI patients. SFC analysis further validated this depolarization phenomenon.

**Conclusion:**

This study revealed MDD patients had suicide risk-specific disruptions of cortical hierarchy, which advance our understanding of the neuromechanisms of suicidality in MDD patients.

## 1. Introduction

Major depressive disorder (MDD) is a common, debilitating, and potentially lethal disorder (1). Patients with MDD usually have high suicide risk levels (2), which aggravate public health crises around the world (3). Previous studies offered a three-step theory of suicide [suicidal ideations (SIs), suicidal attempts (SAs), and suicidal death (SD)] and emphasized the progression from SI to SA (4, 5). It was reported that SI is an important precondition for SA (4–6), while SA is a powerful predictor of completed suicide (7). This progressive relationship indicates that SI and SA may be important intervention points for predicting and preventing suicide. Currently, psychological scales and observed clinical characteristics are the most commonly used methods to evaluate the risk levels of suicide in MDD patients (8, 9). However, these methods cannot identify patients with SI and SA objectively and accurately, particularly for those unwilling to disclose their thinking or commit suicidal acts (5, 8). Therefore, it is urgent to find the objective biomarker to discriminate and identify MDD patients with diverse suicide risk.

Resting-state functional MRI (rs-fMRI) had been widely used to investigate MDD patients with diverse suicidal risks from the perspective of regional brain activity (10) and the brain connectome (9), which has presented inspiring results (11, 12). Wagner et al. found SA had lower regional brain function involved in the fronto-parietal regions and the occipital regions relative to health control and MDD patients without SA by using the amplitude of low-frequency fluctuation index (13). Kang et al. reported SA had higher resting-state functional connectivity (RSFC) of the amygdala and the insula and of the superior orbitofrontal area and the middle temporal area than non-suicide attempters; thus they concluded that the RSFC of the amygdala may reflect suicide risk levels (14). From the perspective of intra-and inter-network interaction, Ho et al. found that lower intra-network coherence of all default subnetworks and insula salience network were associated with the higher past-month SI in depressed adolescents (15). These studies present inspiring results regarding the potential linkage between brain activity or connectivity and suicidality in MDD patients. However, most previous studies focused on a single subgroup (SA or SI) (9–11, 14, 15); thus, there is still a lack of comprehensive, translatable understanding for identifying MDD patients with diverse suicide risks, which warrants further research with new approaches.

Recent research in neuroscience has advanced our understanding that depression is associated with dysfunction of cortical hierarchy (16) as well as disruption of information flow from unimodal sensorimotor networks to the transmodal default network (17), rather than of individual systems alone (17, 18). More recently, Xia et al. demonstrated that MDD patients presented dysfunction of the core connectome hierarchy, which had close linkage with gene expression profiles (16). However, the suicide risk-specific alterations of cortical hierarchy as well as the intact of hierarchical information propagation in MDD patients are still uncovered. The stepwise functional connectivity (SFC) analysis, as a complementary simulation approach for gradient analysis (19), has shown that cortical hierarchy can also be understood as a sequence of steps in connectivity space specifically from unimodal sensorimotor networks to the transmodal default network (19–21). Thus, the combination of two approaches in MDD patients with diverse suicide risks, enable us to understand the specific disruption of the unimodal to transmodal processing as well as their sequence of steps in the connectivity space in MDD patients with diverse suicide risk.

To address these gaps, this study aimed to characterize: (i) the specific macroscale unimodal-to-transmodal gradient alterations in MDD with diverse suicide risks and (ii) the sequence impairment of integration from the unimodal sensory networks to the transmodal networks in MDD with diverse suicide risks.

## 2. Materials and methods

### 2.1. Subjects

Ninety-two adult patients with a diagnosis of MDD (average age: 32.79 ± 12.21 years old; 34 males and 58 females) according to the DSM-IV criteria (22–25) and 38 healthy adults (average age: 37.12 ± 12.61 years old, 13 males and 25 females) with no history of neurological or psychiatric illness participated in this study. Details of inclusion criteria were provided in Supplementary material.

MDD patients were further divided into three subgroups according to their levels of suicide risk, which were estimated by the third item on the HAMD-17 (13, 26, 27). The low suicide risk group [not suicidal (NS) group, *n* = 30] consisted of patients with scores ≤1, indicating the absence of suicidal ideation or suicide attempts within last 2 weeks; the high suicide risk group (*n* = 62) consisted of patients with scores of 2, 3, or 4. The high suicide risk group was then divided into the suicide ideation (SI, *n* = 30) group (with scores of 2 or 3), indicating the presence of suicidal ideation but no suicide attempts within last 2 weeks and the suicide attempt (SA, *n* = 32) group (with scores of 4), indicating a history of suicide attempts within last 2 weeks.

The local Research Ethics Committee approved this study, and written informed consent was obtained from each participant before the study.

### 2.2. Data acquisition and data preprocessing

All of the structural and rs-fMRI images were acquired on a 3.0 Tesla MR scanner (GE Discovery 750) with an eight-channel head coil. Data were preprocessed using DPABI v5.1^1^ (28) following previous studies (20, 29). Detailed imaging parameters and data preprocessing were provided in Supplementary materials.

### 2.3. Connectome gradient analysis

Connectome gradient analysis had been developed to analyze the cortical hierarchy at the systems level, which anchors the unimodal sensory networks on the one end and the transmodal network on the other end (30, 31) and supports the transition from perception to more abstract cognitive functions (31, 32). Gradient mapping provides us a simplified representation in terms of main dimensions to characterize the macroscale cortical hierarchy (16, 31). The connectome gradient analysis was processed using the BrainSpace toolbox ^2^ (33). More details can be found in the Supplementary materials and a previous study (20).

### 2.4. Stepwise connectivity estimation

Stepwise functional connectivity (SFC) analysis is a graph theory-based method that counts the number of defined seeds to other brain regions in a given length of connectivity distance (19–21). More details for stepwise connectivity analysis can be found in the Supplementary materials.

## 3. Statistical analysis

One-way ANOVA and the Chi-square test were performed to detect the differences in demographic and clinical data among the four groups using IBM SPSS v.25.0 (34). *Post hoc* tests were conducted using the Turkey method and Bonferroni method. The significance level was set at *p* < 0.05.

A one-sample *t*-test was performed for SFC to explore the spatial transition patterns of each group. The significance level was set at *p* < 0.0001, FDR corrected.

General linear models implemented in the DPABI toolbox were performed to determine differences between any two groups (HC vs. MDD, MDD-NS vs. MDD-SI, MDD-NS vs. MDD-SA and MDD-SI vs. MDD-SA), including normalized gradient scores and SFC degree at each link-step distance. Sex, age, and education years were set as covariates. Duration of depressive episodes and HAMD score were set as additional covariates in the comparison of patients. The Gaussian random field (GRF) was used for multiple comparison correction (voxel level of *p* < 0.01, cluster level of *p* < 0.05).

## 4. Results

### 4.1. Demographic and clinical characteristics

No significant differences were found in education among healthy controls and MDD subgroups (one-way ANOVA, *F* value = 1.75, *p* = 0.160). However, sex and age differed significantly among them (sex, Chi-square test, *χ^2^* value =11.56, *p* < 0.05; age, one-way ANOVA, *F* value = 4.45, *p* < 0.05). *Post hoc* analysis revealed that the SA group had a higher proportion of women than the NS group and SI group (*p* < 0.05), while the HC group and NS group were older on average than the SI group and SA group (*p* < 0.05). Among MDD patients, the NS group had a longer duration of depressive episodes than the SI group and SA group (*p* < 0.05), while the SA group had a higher HAMD score than the NS group and SI group (*p* < 0.05). The demographic and clinical characteristics information is provided in Table 1.

**Table 1:** Demographic characteristics of controls and MDD patients.

Variables	HC (*N* = 38)		NS (*N* = 30)		SA (*N* = 32)		SI (*N* = 30)		*F or χ^2^* value	*p* value	*post-hoc test*
	Mean	S.D.	Mean	S.D.	Mean	S.D.	Mean	S.D.			
Gender (male: female)	13:25	–	17:13	–	5:27	–	12:18	–	11.56^#^	0.010	NS and SA, SA and SI
Age (years)	37.12	12.61	38.40	11.81	29.66	12.56	30.53	10.54	4.45^*^	0.005	HC > SA, HC > SI, NS > SA, NS > SI
Education (years)	14.89	3.68	13.10	4.36	13.50	3.26	14.13	2.13	1.75^*^	0.160	–
Duration of depressive episodes (years)	–	–	8.11	9.74	3.95	4.79	4.12	4.55	3.61^*^	0.030	NS > SA, NS > SI
HAMD score	–	–	19.40	3.90	24.53	5.29	19.97	3.22	10.04^*^	<0.001	SA > NS, SA > SI
HAMD 3rd item (suicide)	–	–	0.70	0.47	2.60	0.50	4.00	0	522.46^*^	<0.001	SA > SI > NS

HC, health controls; NS, no suicidal group; SI, suicide ideation group; SA, suicide attempt group.

# *χ*^2^, value by Chi-square test.

* The *F* value by One-way ANOVA.

### 4.2. Gradient alterations in MDD with diverse suicide risks

**Figure 1:**
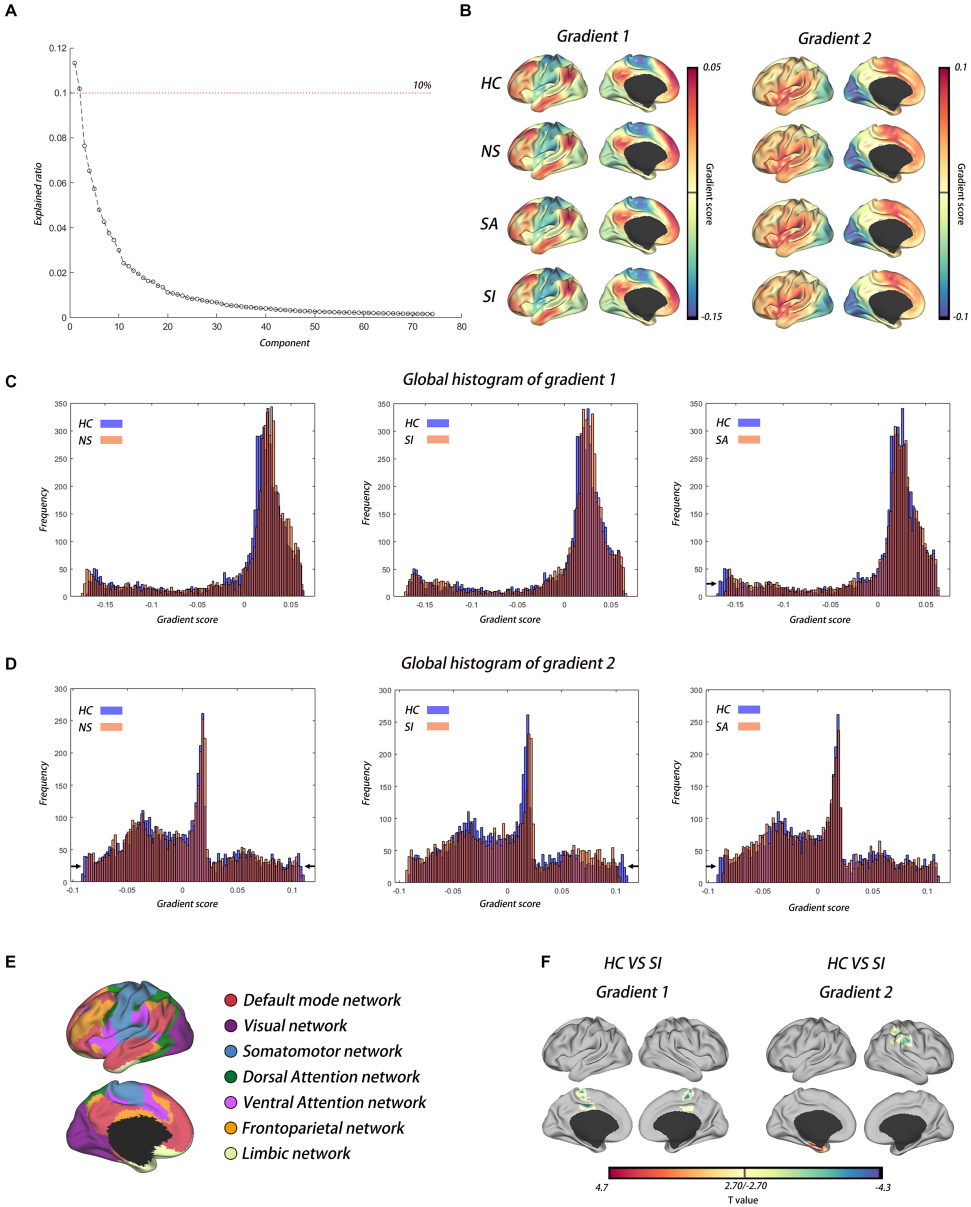
The cortical hierarchy dysfunction in MDD with diverse suicide risks. **(A)** Variance explained by gradient; the dotted curve shows gradients that explained more than 10% of connectome variance in the dataset. **(B)** The first and second functional gradients’ spatial pattern in HC and MDD with diverse suicide risks. **(C,D)** Global histograms showed the depolarization phenomenon in MDD patients with diverse suicide risks compared with HC in gradient 1 and gradient 2, respectively. **(E)** Seven networks template estimated by Yeo. **(F)** Voxel-wise statistical comparisons between HC and MDD patients with SI in gradient 1 and gradient 2, respectively (GRF corrected, voxel level of *p* < 0.01, cluster level of *p* < 0.05). HC, health controls; NS, not suicidal group; SI, suicide ideation group; SA, suicide attempt group.

To maximize interpretability, we only retained principal gradients explaining more than 10% of connectome variance in the dataset following previous studies (20, 21). The first and second principal gradients explained 11.33 and 10.18% of the connectome variance in our dataset, respectively (Figure 1A). The connectome gradient maps for four groups are shown in Figure 1B. The spatial patterns of connectome gradient maps were outstandingly similar among healthy controls and the three MDD subgroups, with the unimodal sensory networks (VIS, AUD, SMN) on the one end and the transmodal network [default mode network, (DMN)] on the other end.

Visual inspection of the global histogram of the principal gradients indicated that the extremes of the unimodal-to-transmodal gradient were contracted uniquely in MDD patients with diverse suicide risks compared with healthy controls. The SA group had a prominent compression from the sensorimotor system of the cortical hierarchy (gradient 1 and gradient 2), the SI group had a prominent compression from higher-level systems such as the frontoparietal network (FPN) and DMN (gradient 2), and the NS group had a compression from both the sensorimotor system and higher-level systems (gradient 2), although it was less prominent than that of patients with SI or SA (Figures 1C,D and Supplementary Figures S1A,B). Group comparison indicated that SI had a higher gradient score in the SMN (e.g., Cingulum_Mid_L) than in the HC (Gradient 1), while SI had a higher gradient score in the DMN (e.g., SupraMarginal_R) and a lower gradient score in the limbic network (e.g., ParaHippocampal_L) than in the HC (Gradient 2) (Figure 1F and Table 2). Compared to NS, SI had a higher gradient score in the VIS (e.g., cuneus), and SA had a lower gradient score in the DMN (e.g., medial frontal gyrus, precuneus) (Supplementary Figure S1D and Table 2). Of note, the whole-brain network allocations were based on the seven networks template by Yeo et al. (35) (Figure 1E).

**Table 2:** Group differences in macroscale functional gradient.

Groups	Gradient	Network	Brain regions	MNI coordinates			*T* value	Voxels
				X	Y	Z		
*HC vs SI*	gradient1	Somatomotor network	Cingulum_Mid_L	−24	−24	60	−4.1708	38
	gradient2	Limbic network	ParaHippocampal_L	−24	−12	−30	3.996	21
		Default mode network	SupraMarginal_R	42	−36	42	−4.286	21

*NS vs SA*	gradient2	Default mode network	Medial Frontal Gyrus	0	66	6	4.682	43
		Default mode network	Precuneus	6	−54	18	4.625	25

*NS vs SI*	gradient2	Visual network	Cuneus	−24	−66	12	−3.5873	92

HC, health controls; NS, no suicidal group; SI, suicide ideation group; SA, suicide attempt group; L, left side of brain; R, right side of brain.

### 4.3. Integration impaired in MDD with diverse suicide risks

**Figure 2:**
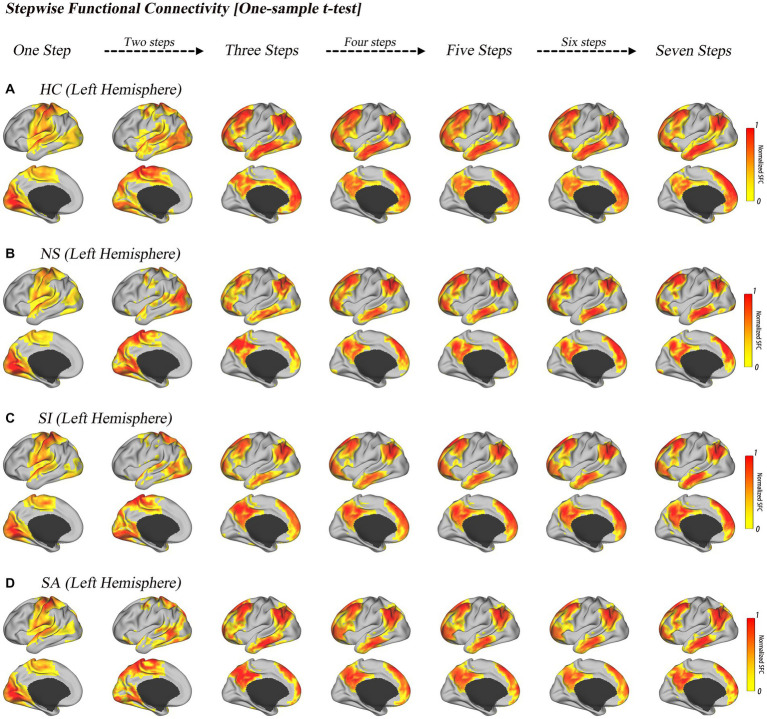
Stepwise functional connectivity pattern in HC and MDD with diverse suicide risks. Surface projections of the one-sample *t*-test results of the unimodal-to-transmodal link-step distances for HC **(A)**, NS **(B)**, SI **(C)**, and SA **(D)**. All of the results are displayed after multiple comparison correction (*p* < 0.0001, FDR corrected). The color bar represents the normalized color scale, 0 represents non-significant results, and 1 is the maximum value corresponding to the smallest *p* value.

Similar spatial transition patterns of stepwise connectivity were observed among healthy controls and the three MDD subgroups (Figure 2). Unimodal sensory-driven networks reached and stabilized at the transmodal networks (DMN) through seven steps (Figure 2).

**Figure 3:**
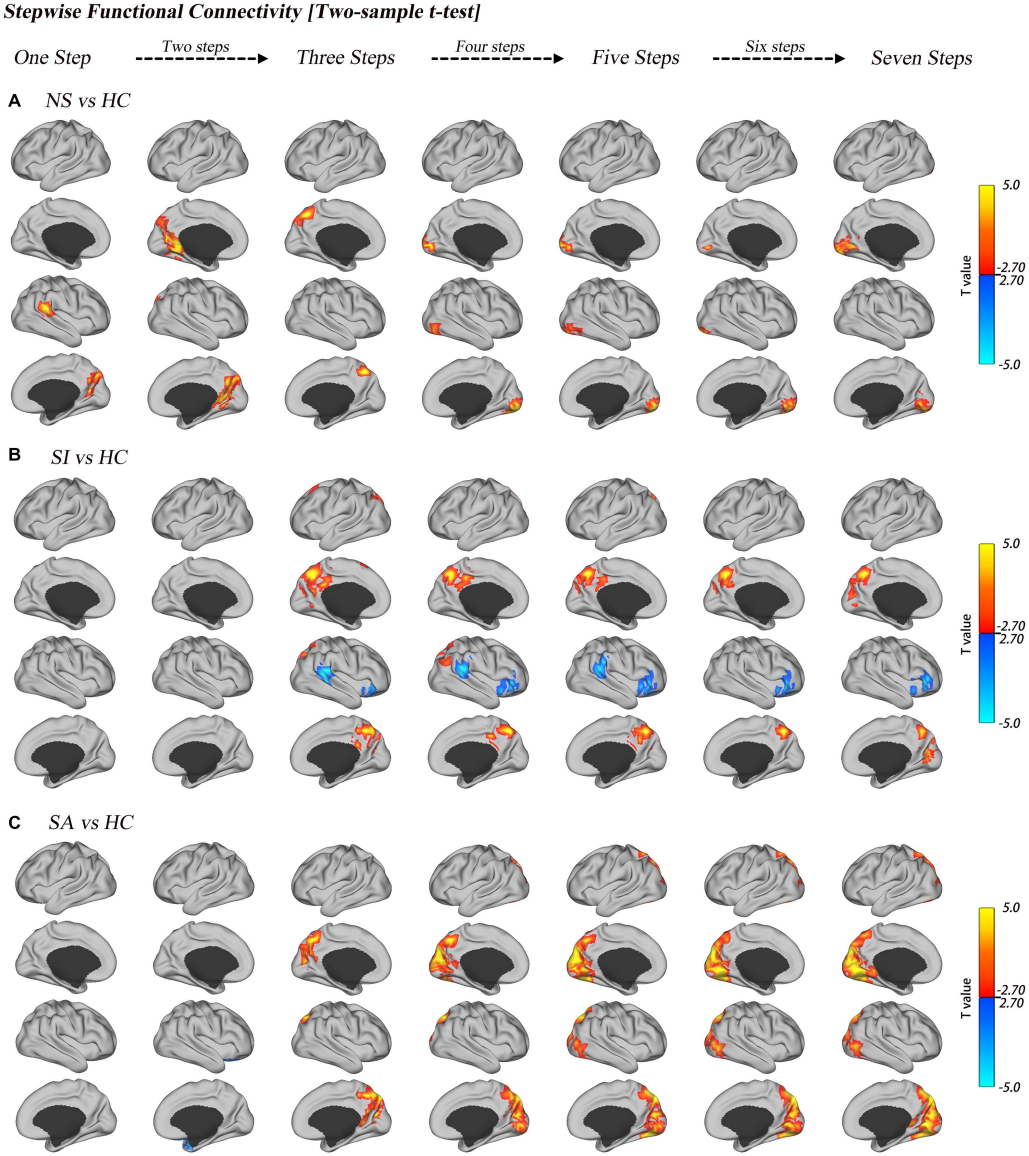
Group comparisons of link-step distance between HC and MDD with diverse suicide risks. Surface projections of the two-sample *t*-test results of the unimodal-to-transmodal link-step distances for NS vs. HC **(A)**, SI vs. HC **(B)**, and SA vs. HC **(C)**. All of the results are displayed after multiple comparison correction (GRF correction, voxel level of *p* < 0.01, cluster level of *p* < 0.05).

Relative to HC, NS patients had an increased SFC degree of the DMN (e.g., precuneus, SupraMarginal_R) at steps 1–3 and increased SFC degree of VIS (e.g., lingual gyrus) at steps 4–7; SI patients had a decreased SFC degree of the limbic network (inferior frontal gyrus and SupraMarginal_R) as well as increased SFC degree of the DMN (precuneus); SA patients had an increased SFC degree of the VIS (e.g., cuneus, Calcarine_L) at steps 3–7, which also extended to the DMN (e.g., precuneus); a transient decrease in the SFC degree of the limbic network (e.g., inferior frontal gyrus) was also observed in step 2. More details are provided in Figure 3 and Table 3.

**Table 3:** Group differences in degree of stepwise functional connectivity.

Groups	Number of step	Network	Brain regions	MNI coordinates			*T* value	Voxels
				X	Y	Z		
*NS vs HC*	One step	Default mode network	Precuneus	18	−72	36	4.42	16
		Default mode network	SupraMarginal_R	60	−42	24	4.07	10
	Two steps	Default mode network	Precuneus	−12	−54	0	5.09	86
	Three steps	Default mode network	Precuneus	0	−60	54	4.68	28
	Four steps	Visual network	Lingual_R	24	−78	−12	4.05	33
	Five steps	Visual network	Lingual_R	48	−66	−12	4.35	37
	Six steps	Visual network	Lingual_R	18	−78	−12	3.67	26
	Seven steps	Visual network	Lingual Gyrus	−12	−90	−12	4.40	43
*SI vs HC*	Three steps	Limbic network	Inferior Frontal Gyrus	42	30	−6	−3.89	12
		Default mode network	Precuneus	0	−72	48	5.56	50
		Default mode network	SupraMarginal_R	60	−36	18	−3.21	14
		Default mode network	Frontal_Mid_L	−36	30	54	3.45	14
	Four steps	Limbic network	Inferior Frontal Gyrus	42	30	0	−4.42	21
		Default mode network	SupraMarginal_R	66	−42	30	−3.92	18
		Default mode network	Precuneus_L	−6	−66	48	5.12	80
	Five steps	Limbic network	Inferior Frontal Gyrus	42	30	0	−4.26	29
		Default mode network	SupraMarginal_R	48	−42	24	−3.87	15
		Default mode network	Precuneus	−6	−66	48	4.92	56
	Six steps	Limbic network	Inferior Frontal Gyrus	42	18	−6	−3.85	28
		Default mode network	Precuneus	−6	−60	48	4.25	41
	Seven steps	Limbic network	Inferior Frontal Gyrus	42	30	0	−3.89	21
		Visual network	Calcarine_R	0	−84	18	3.14	17
		Default mode network	Precuneus	−6	−60	48	4.50	33
*SA vs HC*	Two steps	Limbic network	Inferior Frontal Gyrus	24	6	−30	−3.84	15
	Three steps	Default mode network	Precuneus	−6	−72	48	4.00	90
	Four steps	Default mode network	Precuneus	12	−78	42	4.91	54
		Visual network	Cuneus	0	−65	12	3.91	57
	Five steps	Default mode network	Precuneus	−6	−84	42	4.62	57
		Visual network	Calcarine_L	−7	−73	5	3.66	76
	Six steps	Default mode network	Precuneus	12	−78	42	5.05	54
		Visual network	Calcarine_L	−6	−74	7	3.96	34
	Seven steps	Default mode network	Precuneus	24	−78	48	4.96	62
		Visual network	Calcarine_L	−6	−72	6	4.71	65
*NS vs SA*	One step	Somatomotor network	Postcentral_L	−42	−30	60	−3.74	11
*NS vs SI*	One step	Visual network	Lingual_R	18	−42	−6	3.77	11
		Visual network	Lingual_L	−24	−48	−12	4.26	11
		Somatomotor network	Postcentral_R	18	−36	72	−4.13	10
		Somatomotor network	Postcentral_L	−30	−36	60	−3.63	12
*SI vs SA*	Five steps	Default mode network	Frontal_Sup_Medial_L	0	48	18	3.89	16

HC, health controls; NS, no suicidal group; SI, suicide ideation group; SA, suicide attempt group; L, left side of brain; R, right side of brain.

Pairwise comparison of patients with diverse suicide risks revealed that NS patients had increased SFC degree of the VIS (e.g., Lingual_L, Lingual_R) and decreased SFC degree of the SMN (e.g., Postcentral_L, Postcentral_R) at step 1 when compared to SI patients. NS had decreased SFC degree of the SMN (e.g., Postcentral_L) at step 1 when compared to SA patients. SI patients had increased SFC degree of the DMN (e.g., Frontal_Sup_Medial_L) at step 5 when compared to SA patients. More details are provided in Supplementary Figure S2 and Table 3.

### 4.4. Control analyses

In order to investigate the potential influences of global signal regression (GSR) on gradient analysis and SFC analysis. We further conducted a control analysis without GSR in the functional MRI pre-processing. Furthermore, in order to minimize the risk of Type-I errors or false positives, we re-analyzed the data using a multiple comparison correction threshold of voxel-level *p* < 0.01 and cluster-level *p* < 0.008. Similar results were found between the validation analysis and primary analysis. More details are provided in Supplementary Figures S3–S9 and Supplementary Tables S1, S2.

## 5. Discussion

This is the first study, to our knowledge, to investigate the cortical hierarchy and the sequence of steps in the connectivity space in MDD with diverse suicide risks using connectome gradient and SFC analysis. We demonstrated that a suicide risk-specific disruption of unimodal sensorimotor networks to the transmodal default network existed in MDD patients. Specifically, relative to HC, SA patients had a prominent compression from the sensorimotor system of the cortical hierarchy; SI patients had a prominent compression from the higher-level systems such as the FPN and DMN; NS patients had a compression from both the sensorimotor system and higher-level systems, although it was less prominent than that of SI and SA. The SFC analysis further validated this depolarization phenomenon related to atypical functional transitions from unimodal to multimodal cortical areas in MDD patients with diverse suicide risk levels. Cortical hierarchy disorganization and the sequence of steps in connectivity space abnormalities observed in this study might further improve our understanding of the neural substrates underlying the diverse suicidality in MDD patients.

Hierarchical architecture is one of the recent advances in neuroscience for understanding the organizational principles in the brain (31). First, the unimodal sensory networks (VIS, AUD, SMN) are involved in receiving and abstracting stimulation (36–38). Then, the DMN integrates abstract information with personal emotion, self-cognition, and theoretical ideas and finally guides action in the outside world (39–42). In line with a recent connectome gradient study on MDD (16), this study also observed that MDD patients had compressed global gradient score compared to HC (Supplementary Figure S1A). Moreover, we found the contracted pattern exhibited suicide risk-specific patterns in MDD patients. SA mainly involves the unimodal sensorimotor networks (VIS), the SI mainly involves the transmodal default network (DMN), and NS involves both the unimodal and transmodal networks but less prominently compared to SI and SA (Supplementary Figures S1A,B).

### 5.1. MDD patients with SA exhibited abnormal cortical hierarchy organization mainly in the primary network (VIS)

Compared to HC, MDD patients with SA exhibited gradient compression mainly in the primary network (visual network) in both the first and second gradient. Furthermore, the SFC analysis revealed information transition dysfunction across all of the link-step distances (steps 2–7), which also mainly involved the visual networks, as well as the DMN. The visual network plays important roles in information feedforward and feedback (43, 44). The contact visual network is helpful for resisting disturbance and making a correct response during tasks (43, 44), while a compromised visual network can lead to misconceptions and incorrect decisions in MDD patients with SA (45, 46). Consistent with present results, Holmes et al. indicated that suicide-related imagery, which resulted from the disruption of the visual network, was an essential factor causing suicidal behavior (47, 48). Wagner et al. found SA exhibited attenuated regional brain function as well as decreased functional connectivity involved in the occipital regions (visual related network), although they also revealed other brain regions abnormalities in SA group when compared to health control and MDD patients without SA (13, 49). Stumps et al. found the connection between the visual network and amygdala to be significantly altered in posttraumatic stress disorder patients with SA but not in the psychiatric control group, and they concluded that the disruption of visual network was an important factor causing SA (9). A prior meta-analysis also demonstrated that the hypoactivation of the visual network (e.g., left superior occipital gyrus) was closely related to suicidal thoughts and behaviors (50). Our findings complement previous literature with that MDD with SA possessed abnormal cortical hierarchy organization within the visual network, this disorganization would lead to SA be more concentrated in negative imagery and be more sensitive with negative imagery than HC (48), which would directly influence the emotional system (51) and finally lead to suicidal behavior (52).

### 5.2. MDD with SI exhibited abnormal cortical hierarchy organization mainly in the transmodal network (DMN)

Compared to HC, MDD with SI exhibited gradient compression mainly in the positive pole, where the transmodal network (DMN) is located. The SFC analysis revealed similar findings, which indicated that the information transition dysfunction was also mainly involved in the DMN. The DMN plays a critical role in ruminative processes (53) and eliminating negative information (54); the abnormal cortical hierarchy organization of the DMN observed in SI patients may be responsible for the failure to eliminate negative information (54). Once depressed patients are deeply embedded in negative emotions, they are more likely to have suicidal thoughts (55). The DMN had also been widely recognized as an important node correlating with the severity of SI (46, 56, 57). Schreiner et al. found that an increased connection between the precuneus and primary sensorimotor regions as well as a decreased connection between the posterior cingulate cortex (PCC) and visual attention regions was associated with greater severity of SI in MDD adolescents (58). In MDD adults with SI, the severity of SI was closely correlated with an ongoing low-frequency BOLD signal in the dorsal PCC, ventral PCC, and dorsal anterior cingulate cortex, as well as the decreased connections among these three regions (59). Thus, it is plausible that abnormal cortical hierarchy organization of the DMN may be an important feature for MDD patients with SI.

### 5.3. MDD with NS exhibited abnormal cortical hierarchy organization in both the unimodal and transmodal networks

Compared to HC, MDD with NS exhibited gradient compression in both the unimodal and transmodal networks, although it was less severe than that of MDD with high suicide risk levels (Supplementary Figures S1A,B). SFC analysis revealed that MDD patients with NS had unique disruption during information transition from unimodal to transmodal networks, and the dysfunction of transmodal networks (DMN) occurred at the early link-step distances, while the dysfunction of the visual network occurred at medium–late link-step distances. Although the significance of dysfunction from unimodal to transmodal networks is not clear, our findings were in line with those of recent studies. Using a large cohort of 2,414 participants (1,276 patients with MDD and 1,138 healthy controls), Xia et al. also demonstrated that patients with MDD exhibited abnormal global topography of the principal primary-to-transmodal gradient, as indicated by a reduced explanation ratio, gradient range, and gradient variation (16). Moreover, they found that focal alterations of gradient scores were mostly in the primary systems involved in sensory processing and in the transmodal systems implicated in high-order cognition (16). Our results also agreed with a recent effective connectivity study by Ray et al. using dynamic causal modeling, also demonstrating that depressed patients had hierarchy organization disruption, including compromises in both the backward connections (high-level network to primary network) and forward connections (primary network to high-level network), which were correlated with depressive symptomatology (17). This widespread dysfunction from the unimodal to transmodal networks would lead to failure in the processing of abstract representations (60) and information integration (61), which are characteristics of MDD (17).

## 6. Limitations

Several limitations should be acknowledged. First, the effect of antidepressant therapy was not included in our study, similar to other clinical imaging studies in the field (14, 62). A longitudinal study with detailed experimental and antidepressant profiling of a cohort of treatment-naive MDD patients is needed in the future. Second, the age, gender, and HAMD score were imbalanced among subgroups of MDD patients. Although we regressed them as covariates to reduce the potential influences, we could not eliminate these effects. In the future, we should recruit matched patients and design a more rigorous experiment to exclude such potential influences (63–65). Third, the sample size of the MDD subgroups was relatively small. Thus, these preliminary results should be validated in the future with large sample. Fourth, we only measured the current suicidal thoughts and behaviors (STBs, within last 2 weeks) of MDD patients. We acknowledge the lifetime history of STBs in the NS group could potentially affect the functional brain networks in MDD patients (66, 67). Therefore, it is important to record the lifetime history of STBs in future study.

## 7. Conclusion

In summary, the present work revealed suicide risk-specific disruptions of cortical hierarchy in MDD patients, which advance our understanding of the neuromechanisms of suicidality in MDD patients.

## Data availability statement

The raw data supporting the conclusions of this article will be made available by the authors, without undue reservation.

## Ethics statement

The studies involving human participants were reviewed and approved by Research Ethics Committee of Kangning Hospital. The patients/participants provided their written informed consent to participate in this study.

## Author contributions

LS, ZX, ZY, CS, LX, XZ, and QY: drafting or revision of the manuscript for content, including medical writing for content, study concept or design, analysis or interpretation of data.

HG: major role in the acquisition of data, study concept and design. All authors contributed to the article and approved the submitted version.

## Funding

This study has received funding by Natural Scientific Foundation of China (grant numbers: 81560283 and 81201084) and Natural Science Foundation of Guangdong (grant numbers: 2020A1515011332, and 2022A1515012503), the Shenzhen Key Medical Discipline Construction Fund (no. SZXK041), and the Shenzhen Fund for Guangdong Provincial High-level Clinical Key Specialties (no. SZGSP013).

## Conflict of interest

The authors declare that the research was conducted in the absence of any commercial or financial relationships that could be construed as a potential conflict of interest.

## Publisher’s note

All claims expressed in this article are solely those of the authors and do not necessarily represent those of their affiliated organizations, or those of the publisher, the editors and the reviewers. Any product that may be evaluated in this article, or claim that may be made by its manufacturer, is not guaranteed or endorsed by the publisher.

## Footnotes

^1^ http://rfmri.org/dpabi

^2^ http://brainspace.readthedocs.io
