# Supplementary material for: Cortical hierarchy disorganization in major depressive disorder and its association with suicidality
Source: Front Psychiatry. 2023 Apr 24;14:1140915. doi: 10.3389/fpsyt.2023.1140915 (PMC10165114; doi:10.3389/fpsyt.2023.1140915)
Supplement: Supplementary file 1 [file Table_1.docx]

**Supplement Table 1. Group differences in macroscale functional gradient without GSR**

| **groups** | **Gradient** | **Network** | **Brain regions** | **MNI coordinates** | | | **T value** | **Voxels** |
| --- | --- | --- | --- | --- | --- | --- | --- | --- |
|  |  |  |  | **X** | **Y** | **Z** |  |  |
| **HC vs SI** | gradient1 | Somatomotor network | Cingulum_Mid_L | -7 | -19 | 35 | -3.724 | 60 |
|  | gradient2 | Limbic network | ParaHippocampal_L | -24 | -19 | -25 | 3.36 | 49 |
|  |  | Default mode network | SupraMarginal_R | 48 | -34 | 44 | -4.213 | 33 |
|  |  | Visual network | Calcarine_L | -6 | -72 | 12 | -2.97 | 37 |
|  |  |  |  |  |  |  |  |  |
| **NS vs SA** | gradient2 | Default mode network | Medial Frontal Gyrus | -6 | 64 | 6 | 4.261 | 90 |
|  |  | Default mode network | Precuneus | 7 | -50 | 19 | 4.604 | 29 |
|  |  | Default mode network | Temporal_Mid_R | 48 | -60 | 18 | 3.8921 | 21 |
|  |  |  |  |  |  |  |  |  |
| **NS vs SI** | gradient2 | Visual network | Cuneus | -24 | -72 | 30 | -3.774 | 158 |

Note: HC, health controls; NS, no suicidal group; SI, suicide ideation group; SA, suicide attempt group; L, left side of brain; R, right side of brain.
