# Supplementary material for: Cortical hierarchy disorganization in major depressive disorder and its association with suicidality
Source: Front Psychiatry. 2023 Apr 24;14:1140915. doi: 10.3389/fpsyt.2023.1140915 (PMC10165114; doi:10.3389/fpsyt.2023.1140915)
Supplement: Supplementary file 2 [file Table_2.docx]

**Supplement Table 2. Group differences in degree of stepwise functional connectivity without GSR**

| **groups** | **Number of step** | **Network** | **Brain regions** | **MNI coordinates** | | | **T value** | **Voxels** |
| --- | --- | --- | --- | --- | --- | --- | --- | --- |
|  |  |  |  | **X** | **Y** | **Z** |  |  |
| **NS vs HC** | One step | Default mode network | Precuneus | 16 | -66 | 28 | 3.76 | 21 |
|  |  | Default mode network | SupraMarginal_R | 61 | -41 | 24 | 4.07 | 12 |
|  | Two steps | Default mode network | Precuneus | 17 | -65 | 29 | 4.24 | 115 |
|  | Three steps | Default mode network | Precuneus | -1 | -61 | 56 | 4.32 | 37 |
|  |  | Visual network | Lingual_R | 12 | -90 | -12 | 3.98 | 25 |
|  | Four steps | Visual network | Lingual_R | 24 | -80 | -9 | 4.04 | 41 |
|  | Five steps | Visual network | Lingual_R | 11 | -84 | -14 | 3.50 | 45 |
|  | Six steps | Visual network | Lingual_R | -6 | -90 | -12 | 3.88 | 48 |
|  | Seven steps | Visual network | Lingual Gyrus | -13 | -88 | -17 | 4.19 | 51 |
| **SI vs HC** | Three steps | Limbic network | Insula_R | 42 | 21 | -8 | -3.89 | 13 |
|  |  | Default mode network | Precuneus_R | 4 | -72 | 61 | 5.56 | 102 |
|  |  | Default mode network | SupraMarginal_R | 54 | -43 | 25 | -3.13 | 15 |
|  |  | Default mode network | Frontal_Mid_L | -27 | 27 | 53 | 3.21 | 17 |
|  | Four steps | Limbic network | Inferior Frontal Gyrus | 42 | 23 | -5 | -4.02 | 25 |
|  |  | Default mode network | SupraMarginal_R | 50 | -41 | 30 | -3.92 | 22 |
|  |  | Default mode network | Precuneus_L | -7 | -62 | 47 | 4.70 | 112 |
|  |  | Frontoparietal network | Frontal_Mid_L | -24 | 24 | 54 | 3.73 | 12 |
|  | Five steps | Limbic network | Inferior Frontal Gyrus | 41 | 30 | 5 | -3.82 | 31 |
|  |  | Default mode network | Precuneus | -7 | -71 | 47 | 4.15 | 59 |
|  | Six steps | Limbic network | Inferior Frontal Gyrus | 44 | 31 | 6 | -3.45 | 31 |
|  |  | Default mode network | Precuneus | -8 | -61 | 48 | 4.24 | 45 |
|  |  | Visual network | Calcarine_R | 0 | -84 | 0 | -3.49 | 18 |
|  | Seven steps | Limbic network | Insula_R | 35 | 31 | 0 | -3.207 | 20 |
|  |  | Default mode network | Precuneus_R | 4 | -66 | 46 | 4.02 | 33 |
| **SA vs HC** | Two steps | Limbic network | ParaHippocampal_R | 26 | 7 | -30 | -3.36 | 16 |
|  | Three steps | Default mode network | Precuneus | -2 | -78 | 41 | 3.85 | 107 |
|  |  | Limbic network | Insula_L | -36 | 24 | -12 | -4.23 | 18 |
|  | Four steps | Default mode network | Precuneus | -6 | -71 | 47 | 4.27 | 58 |
|  |  | Visual network | Lingual_L | -1 | -65 | 10 | 3.89 | 29 |
|  | Five steps | Default mode network | Precuneus | 11 | -80 | 56 | 4.26 | 61 |
|  |  | Visual network | Fusiform_L | -24 | -70 | -7 | 4.03 | 42 |
|  | Six steps | Default mode network | Precuneus_R | 10 | -79 | 43 | 4.90 | 59 |
|  |  | Visual network | Lingual_L | -18 | -74 | -4 | 4.04 | 30 |
|  | Seven steps | Default mode network | Precuneus | 13 | -76 | 47 | 3.67 | 54 |
|  |  | Visual network | Lingual_L | -6 | -71 | 5 | 4.58 | 30 |
| **NS vs SA** | One step | Somatomotor network | Postcentral_L | -41 | -29 | 60 | -3.53 | 15 |
| **NS vs SI** | One step | Visual network | Lingual_R | 21 | -53 | -14 | 3.40 | 14 |
|  |  | Visual network | Lingual_L | -26 | -53 | -5 | 3.74 | 11 |
|  |  | Somatomotor network | Postcentral_R | 21 | -43 | 64 | -3.92 | 13 |
|  |  | Somatomotor network | Postcentral_L | -33 | -39 | 58 | -3.56 | 14 |
| **SI vs SA** | Five steps | Default mode network | Frontal_Sup_Medial_L | -1 | 50 | 19 | 3.88 | 24 |

Note: HC, health controls; NS, no suicidal group; SI, suicide ideation group; SA, suicide attempt group; L, left side of brain; R, right side of brain.
