# Supplementary material for: Cortical hierarchy disorganization in major depressive disorder and its association with suicidality
Source: Front Psychiatry. 2023 Apr 24;14:1140915. doi: 10.3389/fpsyt.2023.1140915 (PMC10165114; doi:10.3389/fpsyt.2023.1140915)
Supplement: Supplementary file 3 [file Data_Sheet_1.docx]

**SUPPLEMENTARY MATERIALS**

**Supplementary Materials and Methods**

***Inclusion criteria***

MDD patients were recruited from Shenzhen Kangning Hospital and did not receive standardized drug treatment under the guidance of a doctor within 3 months before the MRI scan. The 17-item Hamilton Depression Scale (HAMD-17) was used to estimate the depression severity of patients. The inclusion criteria for MDD patients were as follows: (1) depressive episode with a HAMD-17 total score ≥ 17; (2) age range from 18 to 60 years old; and (3) right-handed. Patients were excluded if they had (1) other psychotic disorders (including bipolar disorder, which was evaluated by two experienced psychiatrists using the Comprehensive Assessment of Symptoms and History (CASH) (Andreasen, Flaum, & Arndt, 1992), the patients would be excluded if they had experienced at least one manic and/or hypomanic episode); (2) a history of alcohol or substance abuse; (3) substantial prior head trauma; (4) a brain tumor; (5) extremely impaired vision; (6) MRI contraindications and (7) received electrical therapy or other physical therapies in the last 3 months before MR scan. Healthy volunteers were recruited via advertisement on social networks or by word of mouth. Inclusion and exclusion criteria for healthy volunteers were the same, except for the presence of depressive episodes.

***Data acquisition and data preprocessing***

All of the structural and rs-fMRI images were acquired on a 3.0 Tesla MR scanner (GE Discovery 750) with an eight-channel head coil. All of the participants were instructed to keep still with their eyes closed but not to fall asleep and not to think of anything during scanning. Resting-state functional MRI images were acquired using echo-planar imaging (EPI) sequence with following parameters: repetition time (TR) = 2,000 ms, echo time (TE) = 30 ms, flip angle = 90°, slice thickness = 3.5 mm without gap, acquisition matrix = 64 × 64, field of view (FOV) = 224 × 224 mm, number of slices = 33 (interleaved), and 240 time points (8 min). High-resolution structural MRI images were acquired using fast-field echo (FFE) three-dimensional T1-weighted (3D-T1WI) sequences with the following parameters: TR = 6.65 ms, TE = 2.93 ms, flip angle = 12°, acquisition matrix = 256 × 256, and number of slices =192 sagittal slices with no inter-slice gap.

Data were preprocessed using DPABI v5.1 (<http://rfmri.org/dpabi>) (Yan, Wang, Zuo, & Zang, 2016) following previous studies (Dong et al., 2021; Martínez et al., 2020): (1) the first 10 volumes were discarded for signal equilibration; (2) slice-timing adjustment and realignment were done for head-motion correction; (3) those participants whose head translation was more than 1.5 mm and/or rotation was more than 1.5° were excluded from analysis; (4) spatial normalization to the Montreal National Institute 152 standard space was performed at 3-mm isotropic voxel resolution; (5) nuisance covariates were regressed out, including head motion parameters and signals of global, white matter, and cerebrospinal fluid; (6) filtering was done using a band-pass filter (0.009 Hz < f < 0.08 Hz); (7) rs-fMRI was smoothed using a Gaussian kernel of 6 mm full width at half maximum; and then (8) down-sampled to 6-mm isotropic voxels to reduce computational demands.

***Connectome gradient analysis***

The connectome gradient analysis was processed using the BrainSpace toolbox (http://brainspace.readthedocs.io)(Vos de Wael et al., 2020). The main steps included (1) computing the voxel-level connectivity matrix and transforming the matrix using Fisher’s *z*-transformed Pearson’s correlations for each participant; (2) retaining only the top 10% of connections per row, with the remaining connections zeroed, including the negative connections; (3) calculating a cosine similarity matrix that captured similarity in connectivity profiles between each pair of voxels; (4) using a diffusion map embedding algorithm to estimate principal gradient components; (5) calculating an average connectivity matrix from all participants as a group-level gradient component template; and (6) aligning each participant gradient to this template using the Procrustes rotation algorithm.

***Stepwise connectivity estimation***

Stepwise functional connectivity (SFC) analysis is a graph theory-based method that counts the number of defined seeds to other brain regions in a given length of connectivity distance (Dong et al., 2021; S.-J. Hong et al., 2019; Sepulcre, Sabuncu, Yeo, Liu, & Johnson, 2012). We computed and transformed the voxel-level connectivity matrix as the first step of connectome gradient analysis. Then, connectivity matrixes were filtered to contain only positive correlations surviving false discovery rate (FDR) correction at the 0.001 level (Dong et al., 2021; Sepulcre et al., 2012). A previous study indicated that depression is associated with the disruption of information flow from unimodal sensorimotor networks to the transmodal default network (Ray, Bezmaternykh, Mel'nikov, Friston, & Das, 2021). Thus, we choose three seeds, which represent the unimodal sensorimotor networks, for the SFC analysis based on previous studies (Dong et al., 2021; S. J. Hong et al., 2019; Ray et al., 2021; Sepulcre et al., 2010). These three seeds confined within the modular borders of primary sensory cortices (Sepulcre et al., 2010), including the visual network (VIS; Brodmann 17, V1; MNI coordinates x, y, z: −14/10 (left/right), −78, 8), auditory network (AUD; Brodmann 22, A1; −54/58, −14, 8), and sensorimotor network (SMN; Brodmann 3, S1; −42/38, −29, 65), were placed in the unimodal sensory networks as previously described (S. J. Hong et al., 2019; Sepulcre et al., 2012), and each of seed areas was defined as a cubic region containing eight voxels. Then, we calculated the SFC degree map using the “findwalks.m” function provided by the Brain Connectivity Toolbox and standardized the value at each step by subtracting the whole-brain mean of an SFC map and dividing by its standard deviation (Carmona et al., 2015; S.-J. Hong et al., 2019; Martínez et al., 2020). We also constrained our SFC analysis to seven link-step distances as previously described (Martínez et al., 2020; Sepulcre et al., 2012).

***Classification analysis based on connectome gradient and stepwise connectivity***

A support vector machine (SVM) was used to further investigate the classification value of the connectome gradient and SFC in MDD patients with diverse suicide risks. First, one-way ANOVA was used to select the optimal gradient, and SFC features, sex, age, and education years were set as covariates. Gaussian random field (GRF) correction was performed for multiple comparison correction (voxel level of *P* < 0.01 and cluster level of *P* < 0.05). Feature normalization was performed to ensure that the contribution of each feature to the final classification was comparable (Ross, Nandakumar, & Jain, 2006; Wang, Shen, Tang, Zang, & Hu, 2012).

Next, a non-linear SVM with a radial basis function (RBF) kernel was used to conduct classification. First, we evaluated the classifier performance between HC and MDD. Furthermore, we evaluated the classifier performance between MDD patients with low suicide risk and patients with high suicide risk and finally between patients with SA and patients with SI in the high suicide risk group. The leave-one-out cross-validation (LOOCV) method was used for the training and testing of SVM classification. The classification accuracy and receiver operating characteristic (ROC) curves were used to evaluate the performance of the classifier.

**Reference**

Andreasen, N. C., Flaum, M., & Arndt, S. (1992). The Comprehensive Assessment of Symptoms and History (CASH). An instrument for assessing diagnosis and psychopathology. *Arch Gen Psychiatry, 49*(8), 615-623. doi:10.1001/archpsyc.1992.01820080023004

Carmona, S., Hoekzema, E., Castellanos, F. X., García-García, D., Lage-Castellanos, A., Van Dijk, K. R., . . . Sepulcre, J. (2015). Sensation-to-cognition cortical streams in attention-deficit/hyperactivity disorder. *Hum Brain Mapp, 36*(7), 2544-2557. doi:10.1002/hbm.22790

Dong, D., Yao, D., Wang, Y., Hong, S. J., Genon, S., Xin, F., . . . Luo, C. (2021). Compressed sensorimotor-to-transmodal hierarchical organization in schizophrenia. *Psychol Med*, 1-14. doi:10.1017/s0033291721002129

Hong, S.-J., Vos de Wael, R., Bethlehem, R. A. I., Lariviere, S., Paquola, C., Valk, S. L., . . . Bernhardt, B. C. (2019). Atypical functional connectome hierarchy in autism. *Nature communications, 10*(1), 1022-1022. doi:10.1038/s41467-019-08944-1

Hong, S. J., Vos de Wael, R., Bethlehem, R. A. I., Lariviere, S., Paquola, C., Valk, S. L., . . . Bernhardt, B. C. (2019). Atypical functional connectome hierarchy in autism. *Nat Commun, 10*(1), 1022. doi:10.1038/s41467-019-08944-1

Martínez, K., Martínez-García, M., Marcos-Vidal, L., Janssen, J., Castellanos, F. X., Pretus, C., . . . Carmona, S. (2020). Sensory-to-Cognitive Systems Integration Is Associated With Clinical Severity in Autism Spectrum Disorder. *J Am Acad Child Adolesc Psychiatry, 59*(3), 422-433. doi:10.1016/j.jaac.2019.05.033

Ray, D., Bezmaternykh, D., Mel'nikov, M., Friston, K. J., & Das, M. (2021). Altered effective connectivity in sensorimotor cortices is a signature of severity and clinical course in depression. *Proc Natl Acad Sci U S A, 118*(40). doi:10.1073/pnas.2105730118

Ross, A. A., Nandakumar, K., & Jain, A. K. (2006). *Handbook of multibiometrics* (Vol. 6): Springer Science & Business Media.

Sepulcre, J., Liu, H., Talukdar, T., Martincorena, I., Yeo, B. T., & Buckner, R. L. (2010). The organization of local and distant functional connectivity in the human brain. *PLoS Comput Biol, 6*(6), e1000808. doi:10.1371/journal.pcbi.1000808

Sepulcre, J., Sabuncu, M. R., Yeo, T. B., Liu, H., & Johnson, K. A. (2012). Stepwise connectivity of the modal cortex reveals the multimodal organization of the human brain. *J Neurosci, 32*(31), 10649-10661. doi:10.1523/JNEUROSCI.0759-12.2012

Vos de Wael, R., Benkarim, O., Paquola, C., Lariviere, S., Royer, J., Tavakol, S., . . . Bernhardt, B. C. (2020). BrainSpace: a toolbox for the analysis of macroscale gradients in neuroimaging and connectomics datasets. *Commun Biol, 3*(1), 103. doi:10.1038/s42003-020-0794-7

Wang, L., Shen, H., Tang, F., Zang, Y., & Hu, D. (2012). Combined structural and resting-state functional MRI analysis of sexual dimorphism in the young adult human brain: An MVPA approach. *Neuroimage, 61*(4), 931-940. doi:<https://doi.org/10.1016/j.neuroimage.2012.03.080>

Yan, C. G., Wang, X. D., Zuo, X. N., & Zang, Y. F. (2016). DPABI: Data Processing & Analysis for (Resting-State) Brain Imaging. *Neuroinformatics, 14*(3), 339-351. doi:10.1007/s12021-016-9299-4
