# Supplementary figures and images for: Cortical hierarchy disorganization in major depressive disorder and its association with suicidality
Source: Front Psychiatry. 2023 Apr 24;14:1140915. doi: 10.3389/fpsyt.2023.1140915 (PMC10165114; doi:10.3389/fpsyt.2023.1140915)

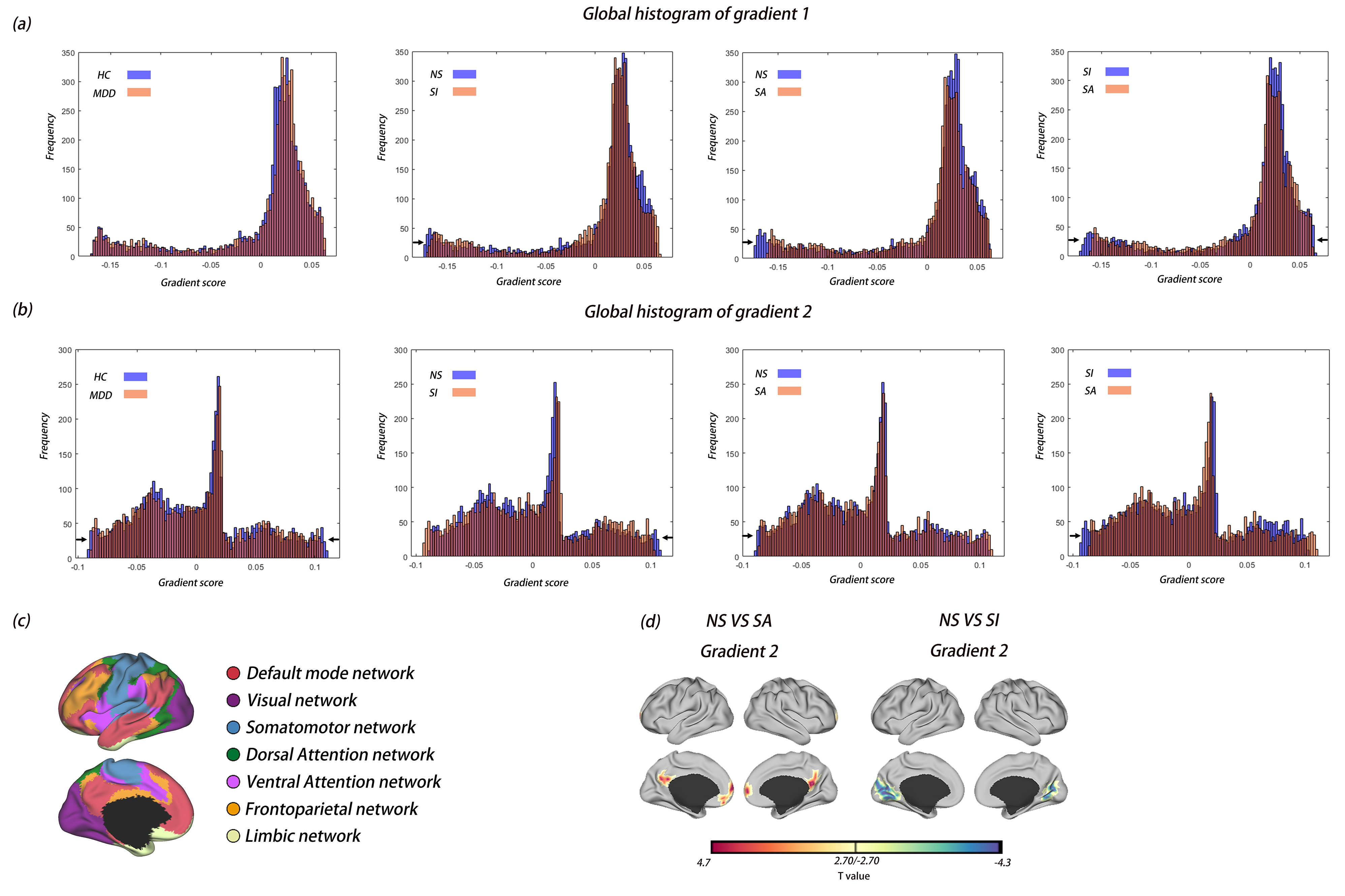

Supplement: Supplementary file 4 [file Image_1.TIF]

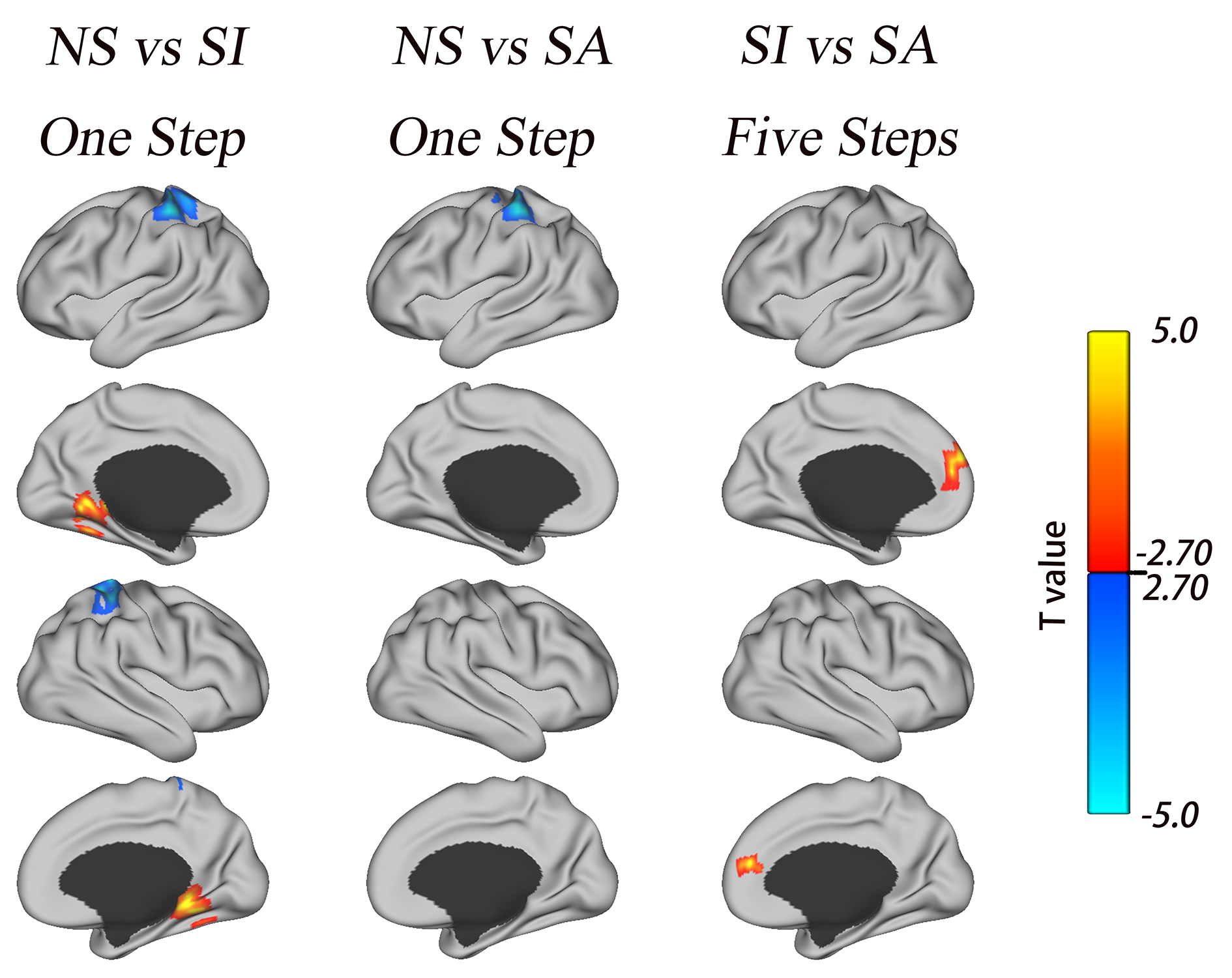

Supplement: Supplementary file 5 [file Image_2.TIF]

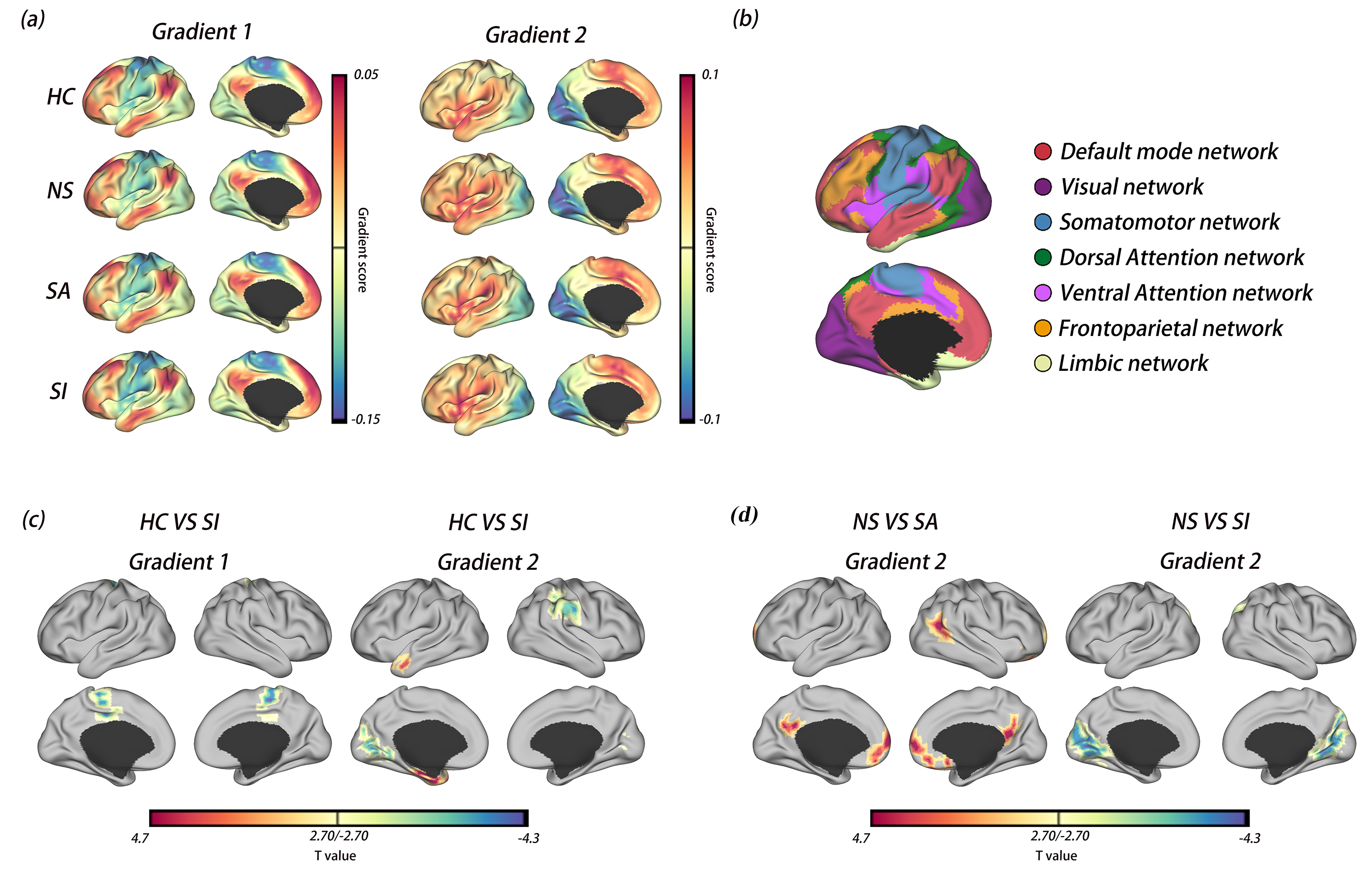

Supplement: Supplementary file 6 [file Image_3.TIF]

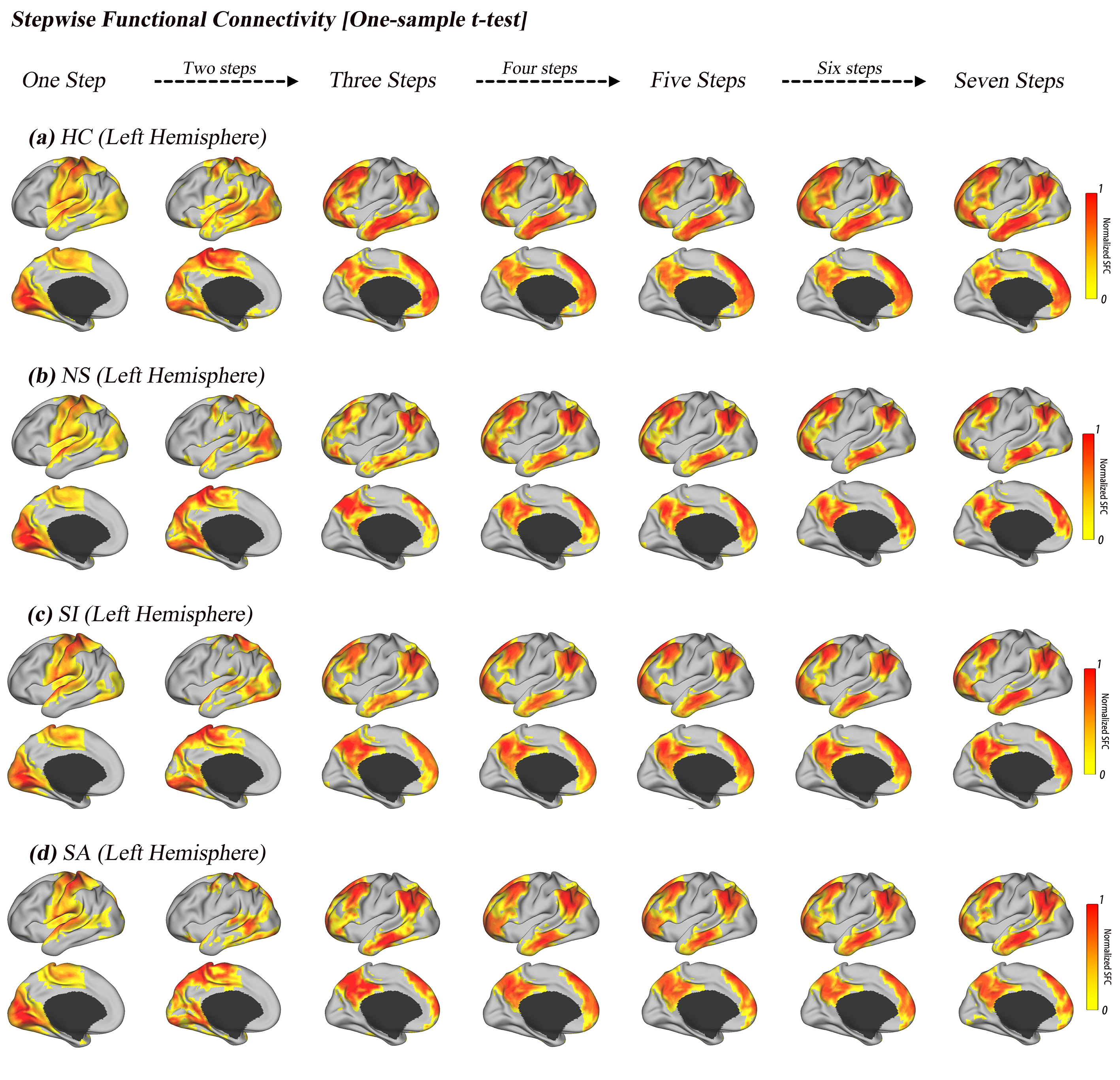

Supplement: Supplementary file 7 [file Image_4.TIF]

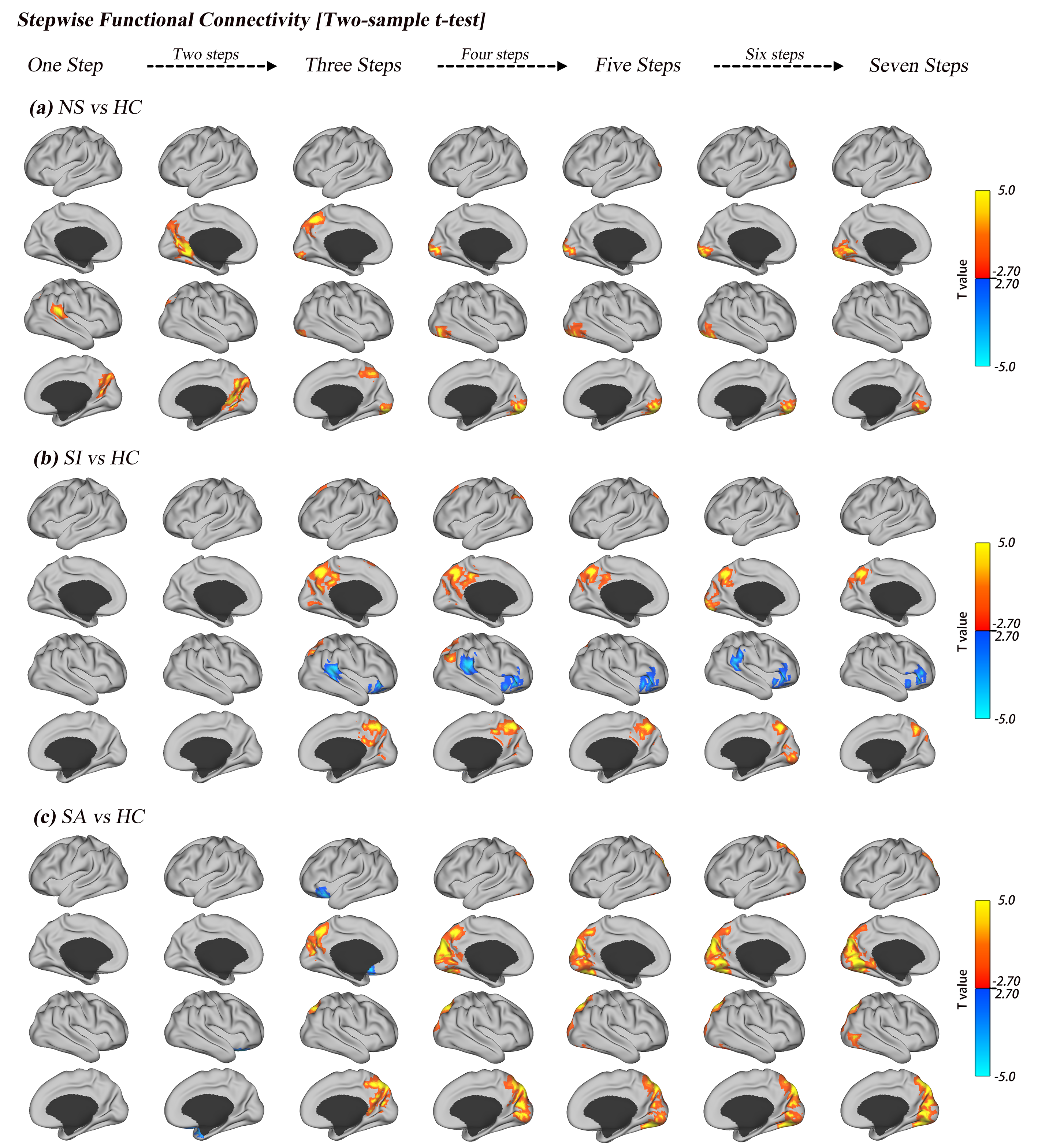

Supplement: Supplementary file 8 [file Image_5.TIF]

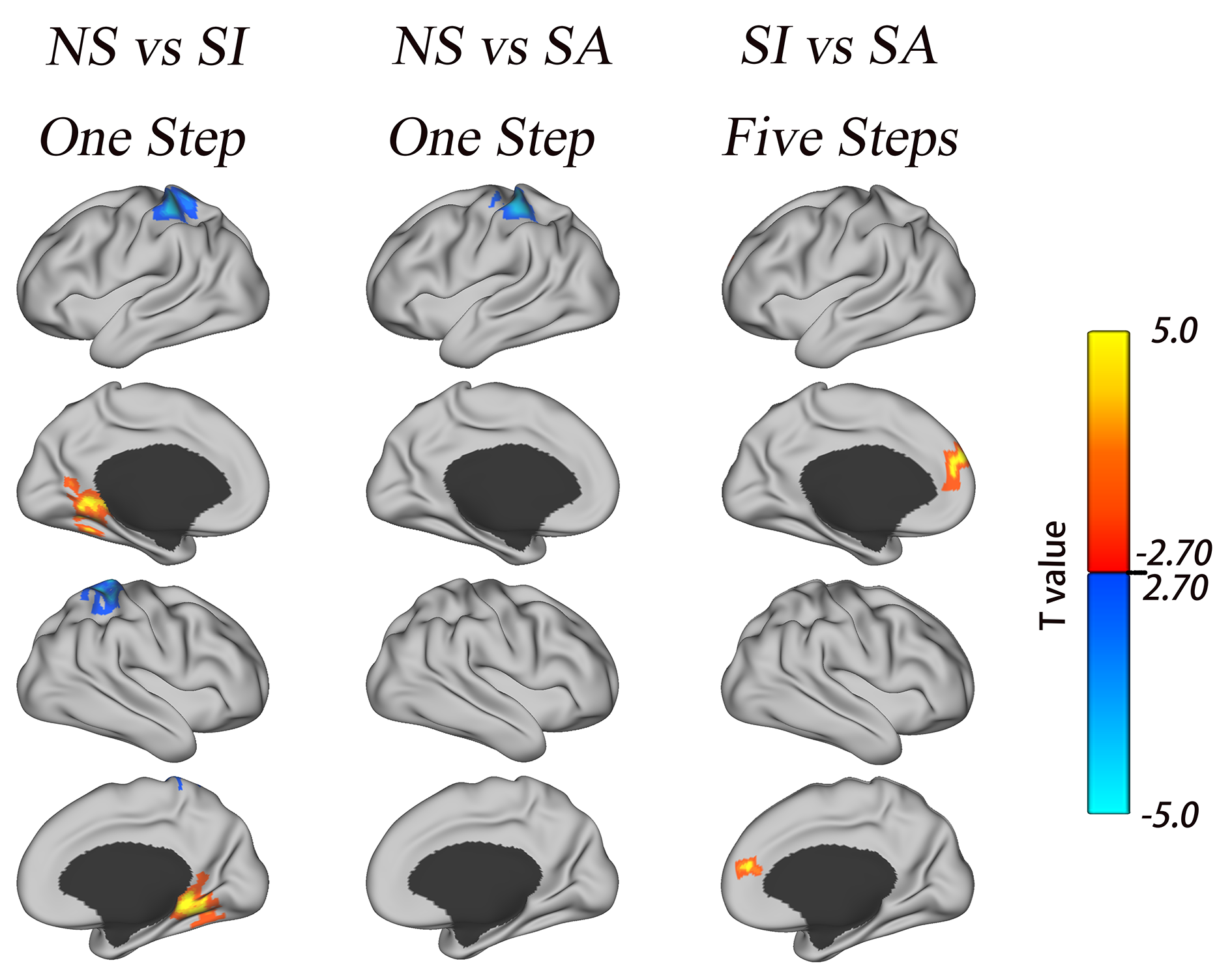

Supplement: Supplementary file 9 [file Image_6.TIF]

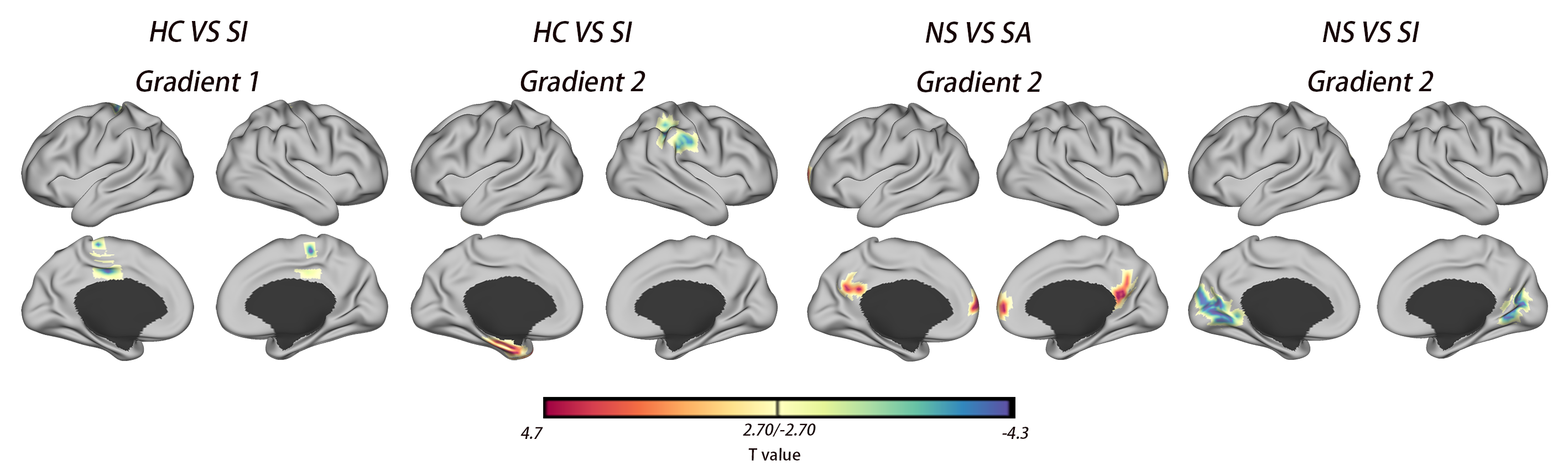

Supplement: Supplementary file 10 [file Image_7.TIF]

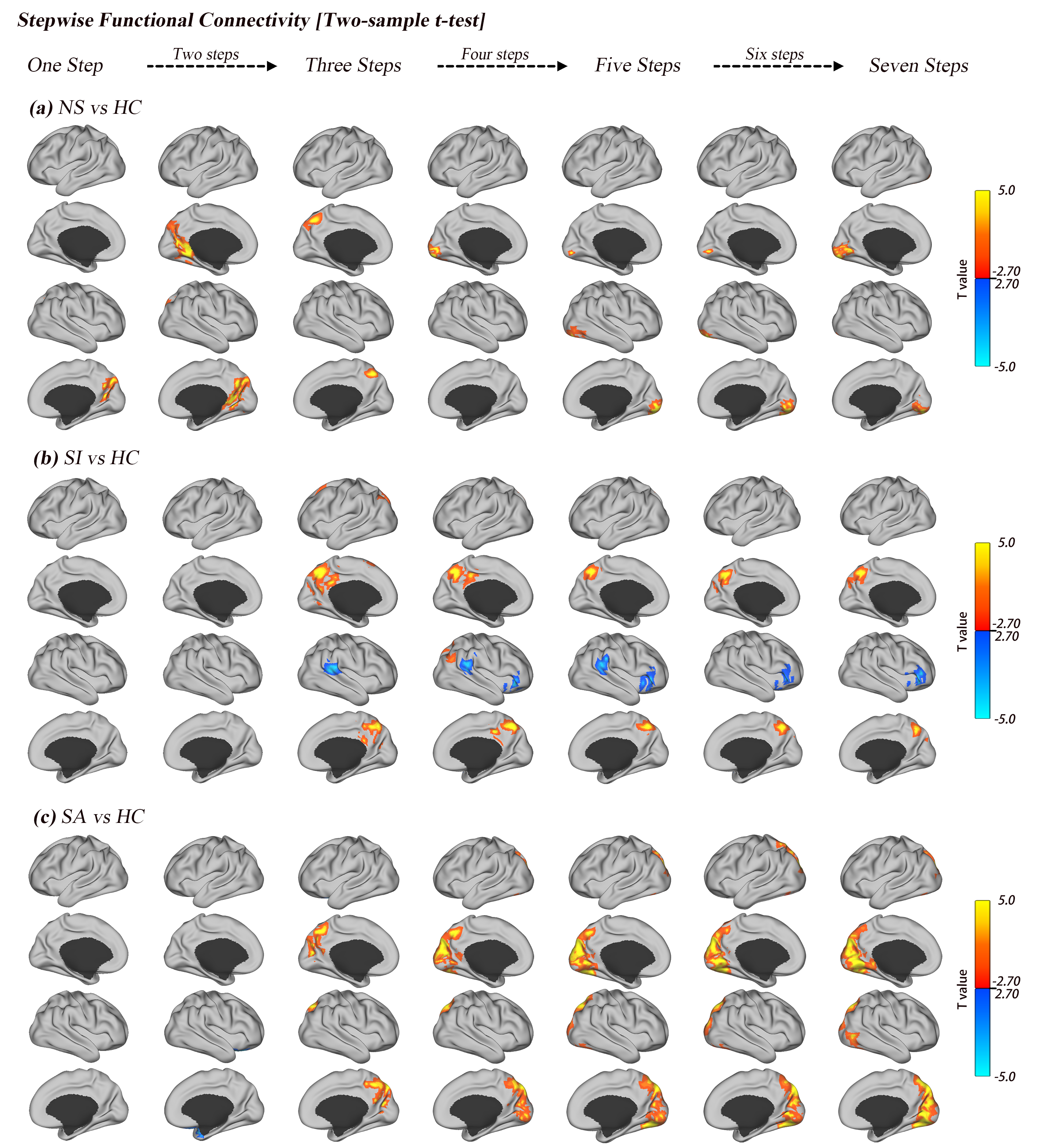

Supplement: Supplementary file 11 [file Image_8.TIF]

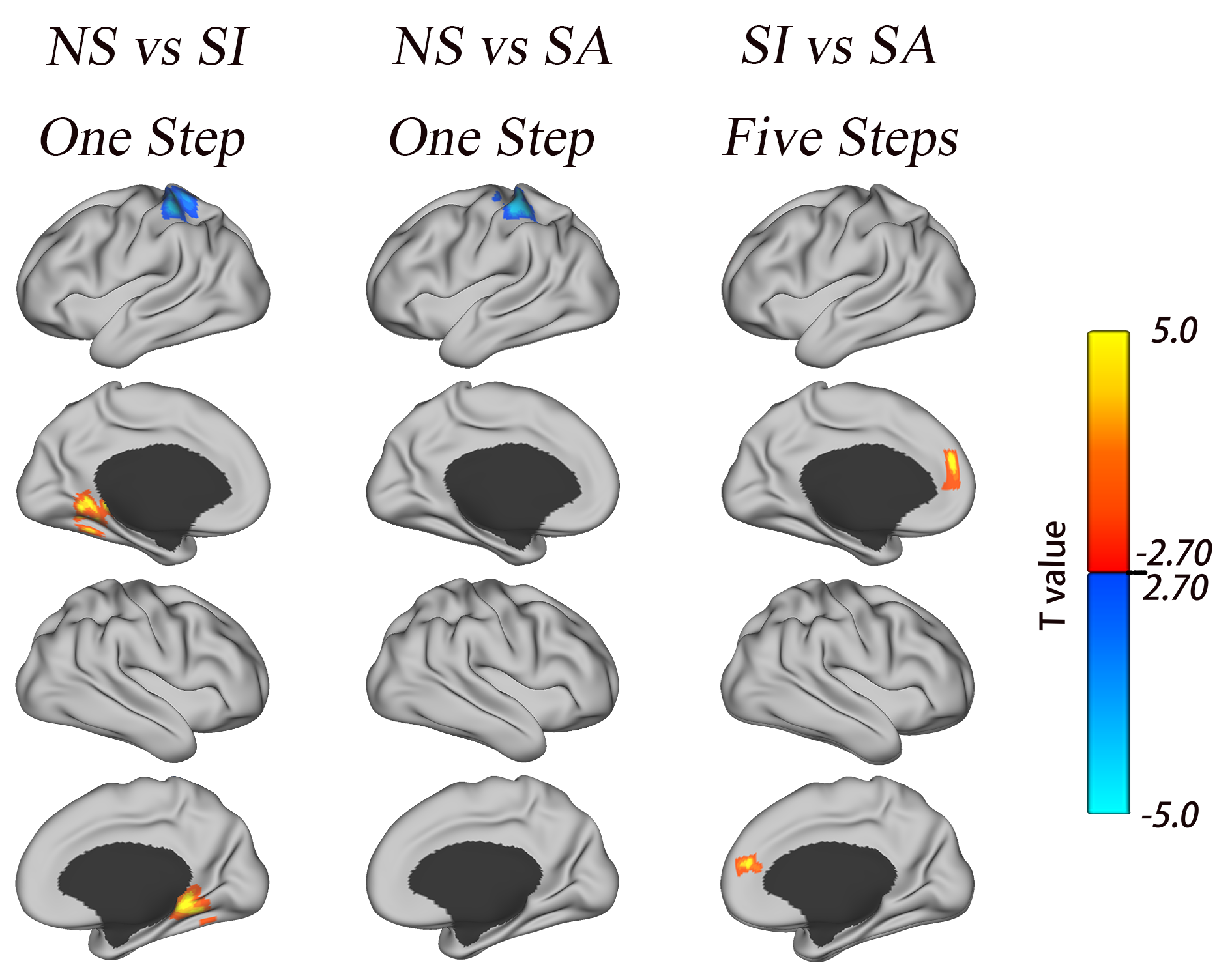

Supplement: Supplementary file 12 [file Image_9.TIF]
